# The Burden of Respiratory Syncytial Virus in Children Under 2 Years of Age in a Rural Community in Maharashtra, India

**DOI:** 10.1093/cid/ciab508

**Published:** 2021-09-02

**Authors:** Ashish Satav, Rowena Crow, Varsha Potdar, Vibhawari Dani, Shilpa Satav, Mandeep Chadha, Danielle Hessong, Phyllis Carosone-Link, Sameer Palaskar, Eric A F Simões

**Affiliations:** 1 MAHAN Trust Mahatma Gandhi Tribal Hospital, Karmgram, Utavali, Tahsil, Dharni, India; 2 Department of Pediatric Infectious Diseases, University of Colorado School of Medicine and Children’s Hospital, Aurora, Colorado, USA; 3 National Institute of Virology, Indian Counsel of Medical Research, Pune, India; 4 Centre for Global Health, Department of Epidemiology, Colorado School of Public Health, Aurora, Colorado, USA

**Keywords:** morbidity, community, epidemiology

## Abstract

**Background:**

Globally, respiratory syncytial virus (RSV) is a common cause of acute lower tract infection (LRTI) in children younger than 2 years of age, but there are scant population-based studies on the burden of RSV illness in rural communities and no community studies in preterm infants.

**Methods:**

Active surveillance of LRTI was performed in the community and hospital setting for the population of 93 tribal villages in Melghat, Central India, over 4 respiratory seasons. A nasopharyngeal swab was obtained from cases presenting as a severe LRTI for molecular analysis of respiratory pathogens including RSVA and B.

**Results:**

High rates of RSV-associated LRTI were found in preterm and term infants beyond 6 months of age, extending into the second year of life. Community severe RSV LRTI rates for 0–11 months of age was 22.4 (18.6–27.0)/1000 child-years (CY) and the hospital-associated rate was 14.1 (11.1–17.8)/1000 CY. For preterm infants, these rates were 26.2 (17.8–38.5)/1000 CY and 12.6 (7.2–22.0)/1000 CY. Comparable rates in the first 6 months were 15.9 (11.8–21.4)/1000 CY and 12.9 (9.3–18.0)/1000 CY in term infants and 26.3 (15.4–45.0)/1000 CY and 10.1 (4.2–24.2)/1000 CY for preterms. The single RSV B season had higher incidences of RSV LRTI in every age group than the 2 RSV A seasons in both preterm and term infants. There were 11 deaths, all term infants.

**Conclusions:**

Studies restricted to the healthcare settings significantly underestimate the burden of RSV LRTI and preterm and term infants have comparable burdens of disease in this rural community.

Respiratory syncytial virus (RSV) is the most common cause of infection of the upper and lower respiratory tract in infants and young children worldwide and is a major public health burden [1, 2]. It has been estimated that globally in 2015 there were 33.1 million episodes of RSV acute lower respiratory tract infections (LRTI) in children younger than 5 years of age, resulting in about 3.2 million hospitalizations [3]. With very few recent studies on the burden of RSV illness in rural communities in lower-middle income countries (LMICs), where most children live [4–10] and given the imminent availability of maternal vaccines and monoclonal antibodies to prevent them [11], there is a need to understand the burden of disease in these rural communities, especially those with high infant mortality.

There are several well-known medical risk groups at higher risk for severe morbidity and mortality from RSV including prematurity [12], chronic lung disease of prematurity [13], congenital heart disease [14], the immune compromised host [15], Down syndrome [16], and other well-defined medical risk groups [17]. Of these, by far the most important and most common is prematurity. A total of 10.6% of the global population of newborn babies are born premature [18], with LMICs having very high rates, where it contributes to the high neonatal and infant mortality, especially in remote rural areas where there is poor access to care [19]. Premature babies have 2- to 3-fold higher rates of hospitalization and intensive care unit stay because of RSV, than do term babies in industrialized nations [12], prompting the development of polyclonal [20] and monoclonal antibodies [21] for its prevention, which have been available in industrialized nations for more than 20 years. There are no recent prospective active surveillance studies from Asia and Africa that have examined the community and hospital burden of RSV in rural areas, comparing the rates of morbidity and mortality in premature and term infants. Therefore, the goal of this study was to compare RSV rates of term and premature infants in Melghat, Maharashtra, an area with high infant mortality (> 50/1000 live births) and frequency of prematurity (13.6%; 11.1–16.1]) [18].

## METHODS

### Study-Site Description Design

This prospective study was conducted in 93 tribal villages of Melghat, Central India. The region is serviced by 2 government and 1 charitable trust (MAHAN) hospitals and 5 primary health centers. Active surveillance for LRTI cases in the community was performed through weekly home visits by village health workers (VHWs). The VHWs were local tribal women living in and accepted by the community. They were trained using standard World Health Organization (WHO) materials, translated into Hindi, with a refresher course every 6 months. A nasopharyngeal (NP) swab was obtained from children with severe or very severe LRTIs and those who died. Samples were tested for a panel of respiratory viruses including RSV.

### Patient Recruitment

Before study startup, clearances were obtained from the Indian Council of Medical Research, Government of Maharashtra, National Institute of Virology Pune, Colorado Multiple Institutional Review Board of University of Colorado, and MAHAN Institutional Review Board. After first seeking permission from village elders, community consent and participation was sought. At that time, a baseline population census of all households in the 93 participating tribal villages was conducted, collecting socioeconomic data for every household.

### Cohort 1: Longitudinal Cohort of Children < 2 Years

After community consent was given, all families identified as having children < 2 years old in the census were approached for consent for study procedures. At enrollment, a detailed parental socioeconomic and demographic questionnaire was administered. Subjects were followed longitudinally until they reached 24 months of age, migrated out of the study villages, or reached the end of the study period.

### Cohort 2: Birth Cohort

Pregnancies in the community were identified by regular home visits to females aged 14 to 50 years by the VHWs, where the date of last menstrual period was recorded. By this method, pregnancies were identified early, and a detailed pregnancy and reproductive history obtained. The VHWs monitored pregnant women, under the supervision of village health supervisors (VHSs), up until delivery, with the newborns enrolled at birth and followed as with the initial longitudinal cohort.

This enrollment strategy gave an initial baseline cohort and ongoing recruitment to a newborn cohort.

### Active Surveillance for LRTIs in the Community

Acute LRTIs were identified through weekly home visits by a VHW assigned to each village. At each visit, the mother was interviewed, and the child assessed for respiratory symptoms. Acute LRTI was determined using standard WHO case definitions [22], with pneumonia diagnosed if there was tachypnea and no chest wall indrawing. Tachypnea is defined as a respiratory rate ≥ 60/min for infants 1 week to 2 months of age, ≥ 50/min for infants 2 to 11 months, and ≥ 40/min for children 12 to 59 months of age. A child with lower chest wall indrawing was classified as having severe pneumonia (with or without tachypnea) and was classified as having very severe pneumonia if there were any danger signs (severe lethargy, difficulty to arouse, or convulsions). If chest wall indrawing or any danger signs were present, a nasopharyngeal sample was obtained. Acute LRTI cases were confirmed by a VHS, who supervised 8–10 of the VHWs.

### Active Surveillance for Medically Attended LRTIs

Medically attended LRTI cases were captured by counsellors assigned at the local health centers and hospitals. Counsellors are locally trained adults, fluent in the local dialect, located in all government hospitals to assist tribal patients. The counsellors assessed every patient in the age group of 0–2 years for LRTIs and obtained nasopharyngeal samples in the same manner as for the community surveillance.

### Mortality Surveillance

VHWs and counsellors monitored all deaths in the villages and in the health facilities. After grief counseling, and obtaining consent from the caregiver, parent and family members, an NP swab was obtained and processed as described later. Fourteen days or so later, the VHSs conducted a verbal autopsy; details are described in the accompanying manuscript [23].

### RSV Testing and Polymerase Chain Reaction Methodology

Nasopharyngeal samples were collected using a flocked swab placed in PrimStore MTM, (Longhorn Vaccines & Diagnostics, Bethesda, MD) for transport to MAHAN hospital, where they were stored at 4–8°C. The PrimStore MTM can be stored at room temperature for 6 months. Samples were transferred to the National Institute of Virology in Pune, India (ICMR) periodically where real-time polymerase chain reaction analysis was performed for a panel of respiratory viruses including RSV [24, 25]. (See Supplemental Material for detailed methodology.)

### Data Analysis

Data were entered into a Microsoft Access database and were rechecked by a data manager. Statistical analysis was performed using Stata statistical software, version 14.2. Analysis was performed on a sample of the newborn cohort where the gestational age (GA) was known. GA was calculated using the birth date and recorded last menstrual period (see Supplemental Material). The WHO classifies newborns as being preterm if birth was less than 37 completed weeks’ gestation (<259 days), which is further divided into 3 categories: moderate or late preterm (GA < 37 weeks and ≥ 32 weeks) very preterm (28-< 32 weeks) and extremely preterm < 28 weeks [26]. All preterm births were combined in our analysis to enable comparison with other population-based studies [18].

The RSV season was determined for each season, starting with the month of the first seasonal case of RSV and ending with the month of the last seasonal case of RSV. Differences in demographics were tested for significance using chi-square and *t* tests. Rates of LRTIs and RSV LRTIs were computed per 1000 child-years (CY) of observation. Ninety-five percent CIs of the rates were computed using the Rothman Greenland Method [27]. For 95% CIs of the incidence rate ratio, the standard error was calculated using the natural log scale to satisfy the normality requirement, and the antilogarithm of the lower and upper confidence limits [28].

Socioeconomic status was estimated using data from the initial parental socioeconomic and demographic questionnaire. A comparable wealth index score was derived from the household’s durable assets and housing characteristics. This summary measure was created using principal components analysis applying factor weights to each variable, with factor weights determined through comparison with the rural subset of the Demographic and Health Survey national survey data for India 2015–2016 (see Supplementary Material for detailed methodology).

## RESULTS

The data presented is from 1 September 2016 to 31 March 2020. The initial baseline cohort consisted of 3676 participants (age 0–24 months). A further 8337 newborns were enrolled throughout the study period with 7110 of the newborns having a calculated GA available. These 7110 newborns were used in this analysis (Supplementary Figure 1).

Classification by GA showed 5997 (84.3%) born at full term with 957 (13.5%) late preterm and 156 (2.2%) early preterm. Demographics of the 3 gestational categories (Table 1) were similar, but showed some significant differences with the early and late preterm subjects more likely to have a nonmedically attended birth and mother less likely to have completed primary school level education and more likely to have no other children <5 years in the household.

**Table 1.** Demographics of Newborn Cohort

	Cohort n = 7110	Term (GA ≥ 37 Weeks)	Late Preterm (GA < 37 ≥ 32 Weeks)	Early Preterm (GA < 32 Weeks)
N	7110	5997 (84.3%)	957 (13.5%)	156 (2.2%)
Male	3656 (51.4%)	3041 (50.7%)	523 (54.6%)	92 (59.0%)
Mean birth weight (g)	2667.5 (SD = 453.3)	(SD = 425.5)	(SD = 499.5)	(SD = 742.7)
Missing birth weight	281 (4.0%)	228 (3.8%)	40 (4.2%)	13 (8.3%)
Birth place				
Home birth	2189 (30.8%)	1802 (30.0%)^a^	330 (34.5%)^a^	57 (36.5%)^a^
Medically attended birth	4907 (69.0%)	4183 (69.8%)	625 (65.3%)	99 (63.5%)
On road/other/not known	26 (0.4%)	20 (0.3%)	3 (0.0%)	3 (0.0%)
Child caretaker				
Parents	7017 (98.7%)	5933 (98.9%)	938 (98.0%)	146 (93.6%)
Grandparents	66 (0.9%)	53 (0.9%)	10 (1.0%)	3 (1.9%)
Other/not known	27.0 (0.4%)	11 (0.2%)	9 (0.9%)	7 (4.5%)
Parents				
Mother’s age (years) mean	23.5 (SD = 453.3)	23.5 (SD = 3.3)	23.5 (SD = 3.3)	23.1 (SD = 3.3)
Mother’s age missing	1 (0.0%)	0 (0.0%)	1 (0.1%)	0 (0.0%)
Mother’s education				
Completed primary	2824 (39.7%)^a^	2331 (38.9%)^a^	431 (45.0%)^a^	62 (39.7%)^a^
More than primary level	4269 (60.0%)	3654 (60.9%)	521 (54.4%)	94 (60.3%)
Not known	17 (0.2%)	12 (0.2%)	5 (0.5%)	0 (0.0%)
Mother’s employment status				
Employed	2034 (28.6%)	1673 (27.9%)	312 (32.6%)	49 (31.4%)
Not employed	5009 (70.5%)	4268 (71.2%)	634 (66.2%)	107 (68.6%)
Not known	67 (0.9%)	56 (0.9%)	11 (1.1%)	0 (0.0%)
Mother’s employment type				
Unskilled labor	1259 (17.7%)^b^	1014 (16.9%)^b^	221 (23.1%)^b^	24 (15.4%)^b^
Other	824 (11.6%)	692 (11.5%)	106 (11.1%)	26 (16.7%)
Not known/not employed	5027 (70.7%)	4291 (71.6%)	630 (65.8%)	106 (67.9%)
Father’s age (years), mean	26.5 (SD = 3.9)	26.5 (SD = 3.8)	26.4 (SD = 4.4)	26.3 (SD = 4.3)
Father’s age missing	1 (0.0%)	0 (0.0%)	1 (0.1%)	0 (0.0%)
Father’s education				
Completed primary	1990 (28.0%)	1653 (27.6%)	289 (30.2%)	48 (30.8%)
More than primary level	5108 (71.8%)	4334 (72.3%)	666 (69.6%)	108 (69.2%)
Not known	12 (0.2%)	10 (0.2%)	2 (0.2%)	0 (0.0%)
Father’s employment status				
Employed	6845 (96.3%)^a^	5793 (96.6%)^a^	902 (94.3%)^a^	150 (96.2%)^a^
Not employed	241 (3.4%)	188 (3.1%)	48 (5.0%)	5 (3.2%)
Other/unknown	24 (0.3%)	16 (0.3%)	7 (0.7%)	1 (0.6%)
Father’s employment type				
Unskilled labor	4008 (56.4%)^a^	3374 (56.3%)^a^	549 (57.4%)^a^	85 (54.5%)^a^
Other	2841 (40.0%)	2422 (40.4%)	355 (37.1%)	64 (41.0%)
Not known/not employed	261 (3.7%)	201 (3.4%)	53 (5.5%)	7 (4.5%)
Household details				
Other children < 5 y				
No other children	3205 (45.1%)^b^	2654 (44.3%)^b^	459 (48.0%)^b^	92 (59.0%)^b^
1 other child	2819 (39.6%)	2398 (40.0%)	375 (39.2%)	46 (29.5%)
2 others	994 (14.0%)	865 (14.4%)	111 (11.6%)	18 (11.5%)
>2 others	91 (1.3%)	80 (1.3%)	11 (1.1%)	0 (0.0%)
Unknown	1 (0.0%)	0 (0.0%)	1 (0.1%)	0 (0.0%)
Other children 5–14 y				
No other children	6304 (88.7%)	5331 (88.9%)	831 (86.8%)	142 (91.0%)
1 other child	529 (7.4%)	446 (7.4%)	74 (7.7%)	9 (5.8%)
2 others	201 (2.8%)	160 (2.7%)	38 (4.0%)	3 (1.9%)
>2 others	75 (1.1%)	60 (1.0%)	13 (1.4%)	2 (1.3%)
Unknown	3 (0.0%)	2 (0.0%)	1 (0.1%)	0 (0.0%)
Wealth score	-1.3 (SD = 1.6)	-1.3 (SD = 1.6)	-1.6 (SD = 1.5)	-1.7 (SD = 1.5)

Abbreviation: GA, gestational age.

^a^*P* < . 01.

^b^*P* < . 001.

**Figure 1.**
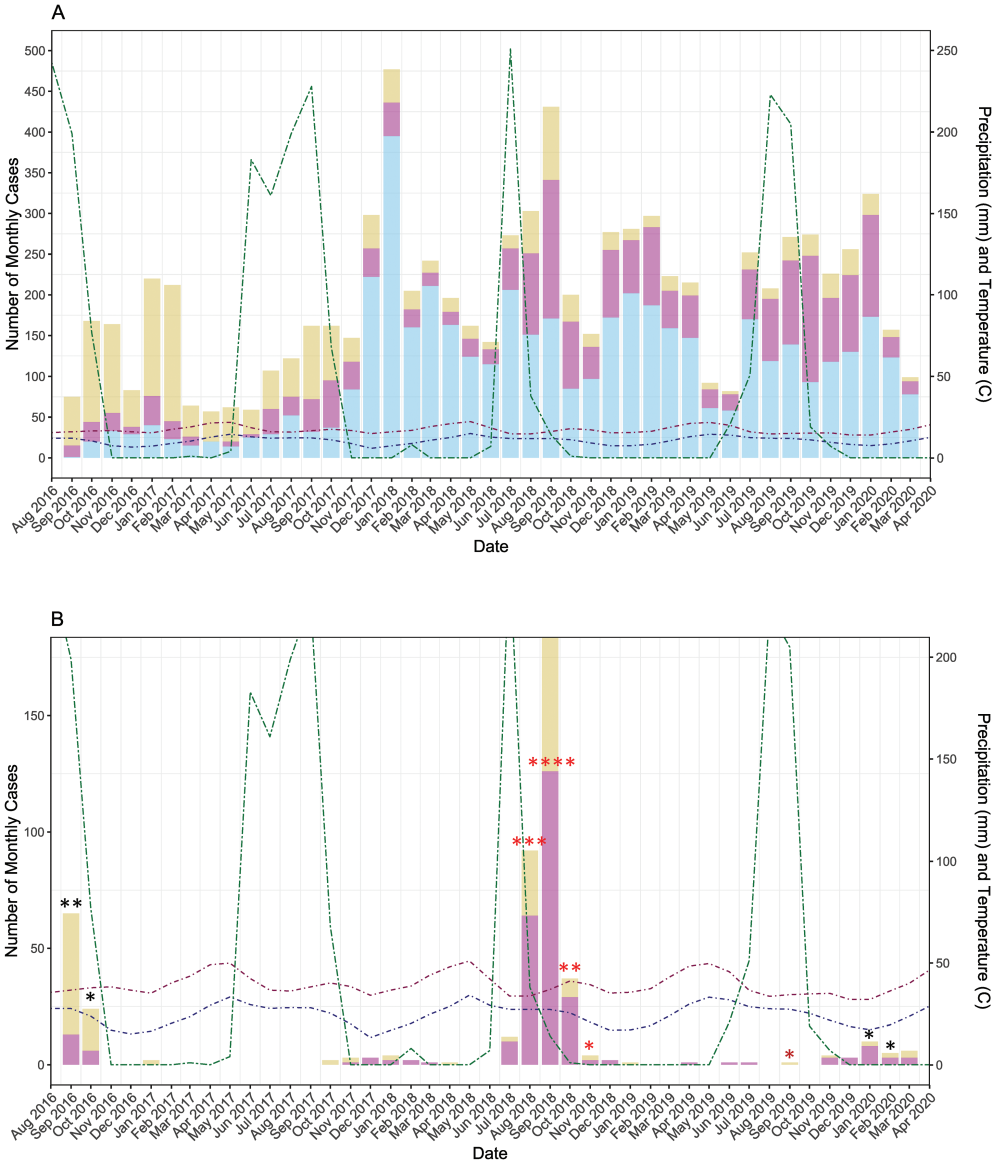
Number of monthly cases of lower respiratory tract infections (LRTIs) from September 2016 through March 2020 in full-term and preterm infants. Monthly total precipitation in millimeters (mm) and monthly mean high and low temperatures in degrees °C are shown to reflect seasonal patterns of infection. (A) Number of monthly full-term cases of severe and very severe LRTIs. (B) Number of monthly preterm cases of severe and very severe LRTIs. (C) Number of monthly full-term cases associated with respiratory syncytial virus (RSV) infection. (D) Number of monthly preterm cases associated with RSV infection.

In term infants, of the 1368 episodes of severe LRTI and 664 episodes of very severe LRTI 181 (13.2%) and 81 (12.2%), respectively, were RSV associated. In preterm infants, these ratios were 34/226 (15.0%) for severe LRTIs and 19/283 (6.7%) very severe LRTIs, respectively. Figure 1A and B show the seasonality pattern of severe and very severe LRTIs for subjects born at full-term and preterm, respectively. Both severe and very severe LRTI numbers were highest during the rainy season (September) when precipitation was high and during the cold season (November–January) and when the temperature is low. There were 2 peaks in RSV LRTI cases in 2016 and 2018 (Figure 1C and D) during the rainy season when precipitation is high, and temperatures are lower. During intermediate years (ie, 2017 and 2019), a small number of RSV LRTI cases are seen.

**Figure 2.**
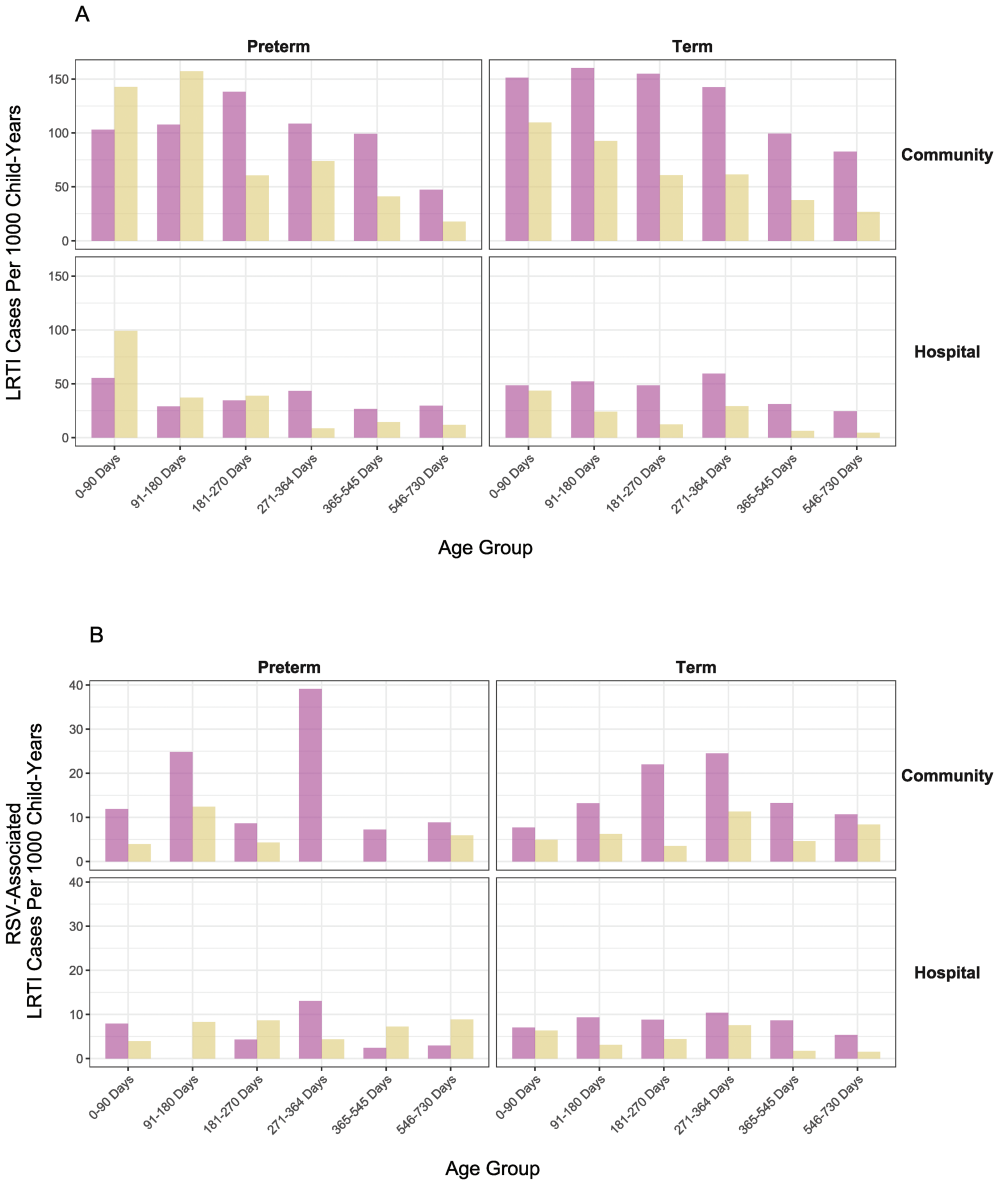
Rates of lower respiratory tract infections (LRTIs) by gestation and age group. (A) Rates of preterm (left) and full-term (right) severe and very severe LRTI in community and hospital, by age group. (B) Rates of preterm (left) and full-term (right) severe and very severe LRTI associated with respiratory syncytial virus (RSV) infection in community and hospital, by age group.

Both the rates of severe and very severe LRTI by age group were found to be comparable in both the term and preterm groups (Figure 2A), with increased rates in the first 6 months of life. Comparing full-term to preterm, the proportion of all very severe LRTI vs severe LRTI is higher in the preterm group (odds ratio [OR]: 1.66, 95% CI: 1.34–2.07; *P* < .0001). Compared with the community rates, hospital rates of severe and very severe LRTI are lower in all age groups, both full-term and preterm.

Community RSV-associated rates for both the severe and very severe LRTI increase from birth to 365 days, and then decline (Figure 2B); the overall rates are similar between preterm and term children (OR, 0.65; 95% CI, 0.26–1.50). In the hospital setting, the rates of very severe RSV-associated LRTIs in the first 2 years of life are higher in the preterm group than term group (OR, 3.15; 95% CI, 1.19–8.27). In the preterm group itself, hospital rates of very severe LRTI are higher than community rates (OR, 5.57; 95% CI, 1.64–18.57), whereas these rates are similar in term infants (OR, 1.11; 95% CI, 0.65–1.90).

In full-term infants, the incidence rate for severe/very severe RSV LRTI for age 0 to 365 days is 22.4 (18.6–27.0) per 1000 CY in the community and 14.1 (11.1–17.8) per 1000 CY in the hospital setting (Table 2). For preterm infants in the community, incidence rates are higher than for full-term infants at 26.2 (17.8–38.5) per 1000 CY, but in the hospital setting incidence rates are lower for preterm infants than for full term at 12.6 (7.2–22.0) per 1000 CY (Table 2).

**Table 2.** Incidence Rate Ratios for Full-term and Preterm RSV-associated Lower Respiratory Tract Infections

	Overall			RSV Season		
	Full-term	Preterm		Full-term	Preterm	
Age	Incidence (95% CI)	Incidence (95% CI)	IRR (95% CI)	Incidence (95% CI)	Incidence (95% CI)	IRR (95% CI)
Days	/1000 C-Y	/1000 C-Y		/1000 CY	/1000 CY	
Community						
0–90	12.7 (8.0–20.0)	15.8 (6.0–41.9)	1.25 (0.37–3.28)	20.1 (12.7–31.8)	25.9 (9.9–68.2)	1.29 (0.38–3.32)
0–180	15.9 (11.8–21.4)	26.3 (15.4–45.0)	1.66 (0.69–2.24)	25.9 (19.3–34.8)	45.9 (27.0–78.1)	1.77 (0.71–2.31)
0–270	18.7 (14.9–23.5)	22.1 (13.6–35.8)	1.18 (0.64–1.80)	39.8 (31.7–49.9)	52.0 (32.3–83.8)	1.31 (0.67–1.88)
0–364	22.4 (18.6–27.0)	26.2 (17.8–38.5)	1.17 (0.71–1.61)	37.0 (30.8–44.5)	48.7 (33.2–71.4)	1.32 (0.75–1.70)
0–545	21.3 (18.0–25.0)	20.4 (14.2–29.5)	0.96 (0.67–1.44)	34.9 (29.7–41.1)	36.3 (25.3–52.3)	1.04 (0.69–1.49)
0–730	20.9 (18.0–24.3)	19.3 (13.8–27.1)	0.92 (0.68–1.38)	34.3 (29.5–39.8)	33.7 (24.1–47.1)	0.98 (0.70–1.41)
Hospital						
0–90	13.4 (8.5–20.9)	11.9 (3.9–36.6)	0.89 (0.28–3.27)	21.2 (13.6–33.1)	19.4 (6.3–59.6)	0.92 (0.28–3.31)
0–180	12.9 (9.3–18.0)	10.1 (4.2–24.2)	0.78 (0.35–2.29)	21.1 (15.2–29.3)	17.7 (7.4–42.1)	0.84 (0.36–2.35)
0–270	13.0 (9.9–17.1)	11.0 (5.5–22.0)	0.85 (0.45–1.94)	27.6 (21.0–36.3)	26.0 (13.1–51.5)	0.94 (0.47–2.03)
0–364	14.1 (11.1–17.8)	12.6 (7.2–22.0)	0.89 (0.53–1.72)	23.2 (18.4–29.3)	23.4 (13.4–40.9)	1.01 (0.55–1.82)
0–545	13.1 (10.6–16.2)	11.7 (7.2–19.0)	0.89 (0.57–1.59)	21.5 (17.5–26.5)	20.8 (12.8–33.7)	0.96 (0.59–1.64)
0–730	12.1 (9.9–14.7)	11.7 (7.6–18.1)	0.97 (0.62–1.56)	19.8 (16.3–24.2)	20.4 (13.2–31.5)	1.03 (0.64–1.60)

Abbreviation: CY, child-years; IRR, incidence rate ratio.

The incidence rate ratio of preterm to full-term RSV-associated LRTI (Table 2) are, for the most part, lower in the hospital setting, contrasting with higher values in the community setting.

While examining RSV-associated LRTI rates by subtypes A and B, we found that all of the RSVA cases in the preterm group were classified as very severe LRTI compared to only 52% of the full-term infants. For RSVB subtype, the proportion of very severe RSV-associated LRTI was 44.8% full-term and 52.8% preterm (Table 3).

**Table 3.** Rates by Child Years of Observation of Respiratory Syncytial Virus Associated Lower Respiratory Tract Infections in Term and Preterm Children

	Term					Preterm				
	CY	Severe RSV LRTI	Very Severe RSV LRTI ^a^	Severe RSV LRTI	Very Severe RSV LRTI	CY	Severe RSV LRTI	Very Severe RSV LRTI	Severe RSV LRTI	Very Severe RSV LRTI
Age (days)		No.	No.	1000 CY (95% CI)	1000 CY (95% CI)		No.	No.	1000 CY (95% CI)	1000 CY (95% CI)
RSV Positive										
0–90	1422.5	21	16	14.8 (22.6–9.7)	11.2 (18.3–6.9)	252.5	5	2	19.8 (47.2–8.3)	7.9 (31.5–2.0)
0–180	2708.5	50	28	18.5 (24.3–14.0)	10.3 (14.9–7.2)	494.2	11	7	22.3 (39.9–12.4)	14.2 (29.6–6.8)
0–270	3844.4	85	37	22.1 (27.3–17.9)	9.6 (13.3–7.0)	725.8	14	10	19.3 (32.4–11.5)	13.8 (25.5–7.4)
0–364	4903.8	122	57	24.9 (29.6–20.9)	11.6 (15.0–9.0)	955.9	26	11	27.2 (39.7–18.6)	11.5 (20.7–6.4)
0–545	6636.8	160	69	24.1 (28.1–20.7)	10.2 (13.1–8.2)	1369.5	30	14	21.9 (31.2–15.4)	10.2 (17.2–6.1)
0–730	7945.0	181	81	22.8 (26.3–19.7)	10.2 (12.7–8.2)	1707.8	34	19	19.9 (27.8–14.3)	11.1 (17.4–7.1)
RSV A positive										
0–90	1422.5	4	4	2.8 (7.5–1.1)	2.8 (7.5–1.1)	252.5	0	1	0.0 (0.0–0.0)	4.0 (28.0–0.6)
0–180	2708.5	8	7	3.0 (5.9–1.5)	2.6 (5.4–1.2)	494.2	0	1	0.0 (0.0–0.0)	2.0 (14.3–0.3)
0–270	3844.4	14	8	3.6 (6.1–2.2)	2.1 (4.2–1.0)	725.8	0	1	0.0 (0.0–0.0)	1.4 (9.8–0.2)
0–364	4903.8	16	8	3.3 (5.3–2.0)	1.6 (3.3–0.8)	955.9	0	1	0.0 (0.0–0.0)	1.0 (7.4–0.1)
0–545	6636.8	19	9	2.9 (4.5–1.8)	1.4 (2.6–0.7)	1369.5	0	1	0.0 (0.0–0.0)	0.7 (5.2–0.1)
0–730	7945.0	20	10	2.5 (3.9–1.6)	1.3 (2.3–0.7)	1707.8	0	1	0.0 (0.0–0.0)	0.6 (4.2–0.1)
RSV B positive										
0–90	1422.5	17	13	12.0 (19.2–7.5)	9.1 (15.7–5.3)	252.5	5	1	19.8 (47.2–8.3)	4.0 (28.0–0.6)
0–180	2708.5	42	22	15.5 (20.9–11.5)	8.1 (12.3–5.4)	494.2	11	6	22.3 (39.9–12.4)	12.1 (26.9–5.5)
0–270	3844.4	71	30	18.5 (23.3–14.7)	7.8 (11.1–5.5)	725.8	14	9	19.3 (32.4–11.5)	12.4 (23.7–6.5)
0–364	4903.8	106	50	21.6 (26.1–17.9)	10.2 (13.4–7.7)	955.9	26	10	27.2 (39.7–18.6)	10.5 (19.4–5.6)
0–545	6636.8	141	60	21.2 (25.0–18.0)	9.0 (11.6–7.0)	1369.5	30	13	21.9 (31.2–15.4)	9.5 (16.3–5.5)
0–730	7945.0	161	72	20.3 (23.6–17.4)	9.1 (11.4–7.2)	1707.8	34	18	19.9 (27.8–14.3)	10.5 (16.7–6.7)

Abbreviations: CY, child-years; RSV LRTI, respiratory syncytial virus lower respiratory tract infection.

^a^1 case with RSVA and RSVB.

There were 11 RSV-associated deaths, 10 in the community and 1 hospital death. All deceased children were born full-term.

## Discussion

This study in a rural part of India with a high infant mortality shows some unexpected findings regarding both the morbidity and mortality from RSV LRTI in preterm infants and young children compared to full-term babies. Although the pattern of LRTIs overall does show that very severe LRTI predominates in preterm infants in the first 6 months of life compared with severe LRTI predominating in term infants, this pattern is not replicated in the burden of RSV LRTI in the community and hospital setting. Second, the overall rates of RSV LRTIs in both the community and in the hospital appear to be similar between preterm babies and full-term babies. Third, RSVB appears to be much more severe in preterm babies than in term infants. Finally, all of the mortality attributed to RSV in this study occurred in full-term infants and young children. There were no RSV-related deaths in preterm babies.

It is clear that, in this community study, the pattern of severe/very severe RSV LRTIs in the community, in both full-term and preterm infants, appears to be highest in the third quarter of the first year, conversely being almost the lowest in the first 3 months of life. This is at odds with most meta-analyses [3, 29] as well as community-based studies of RSV that used hospitalization as the anchoring point and community adjustments for calculating severe RSV LRTI rates in the community summarized in Shi et al.[3]. An earlier study in Indonesia, with weekly home visits conducted over 28 months, also showed a similar pattern of RSV LRTIs in the community [6]. In that manuscript, we discussed at length the various possibilities for that paradoxical observation. Subsequently, other active surveillance studies of infants and young children, although not as large as this study, have also shown that severe RSV LRTI occurs in older children [30–33], though the studies in Kenya [31] and Nicaragua [32] show high rates in the first 2 months of life. In Nicaragua, the incidence of severe RSV LRTI was 21.7 (10.9–43.4)/1000 CY in infants 6–11 months of age, similar to the 0- to 11-month rate in term infants in our study 22.4 (18.6 – 27.0)/1000 CY is for. In a prospective community study in Peru, the rates of RSV LRTI (severity not included) showed similar rates of RSV LRTI 30–34/1000 CY in the first 23 months of life throughout all age groups [34], but only 4% were hospitalized.

At least in the first year, for most of these time periods preterm babies have marginally higher rates of RSV LRTI than term infants overall and in the community; however, hospitalization rates are almost equivalent. Most systematic reviews show rates of RSV hospitalization between 2 and 3 times higher for preterm infants than full-term infants [12]. There are very few studies in LMICs. One study from Peru examined the rates in 222 premature infants < 1500 g with rates of RSV hospitalization of 88/1000 CY for infants 1000–1500 g in the first year of life [35]; however, there was no term infant comparator in that study. A recent estimate of the preterm hospitalization rates in the first year of life was 63.85 (95% CI, 37.52–109.7)/1000 CY [29], more than twice the rate in this study of 26.2 (17.8–38.5)/1000 CY. In contrast, full-term hospitalization rates of 12.9 (9.3–18.0)/1000 CY in 0–5 months is closer to the overall global estimates of 20.2 (16.7–30.2)/1000 CY for developing countries [3] and the rate in the first year of life 14.1 (11.1–17.8)/1000 CY is closer to a recent meta estimate of 11.1 (9.8–12.3)/1000 CY of active surveillance study estimates in the United States [36]. There is also a significant burden of disease into the second year of life in both preterm and full-term children in this population.

For RSV-associated LRTIs, RSV subtype B was found to be more common than subtype A. Curiously, despite small numbers there was only 1 case of RSVA in the preterm population. For RSVB, the point estimates for severe and very severe LRTI in preterm infants was almost always slightly higher than in full-term infants. This study could not differentiate between the sero groups for severity.

Finally and most surprisingly, none of the deaths occurred in preterm infants; all occurred in full-term infants. Because most of these occurred in the community, and there were no ventilators or other source of assisted ventilation available in the health centers and hospitals, it is possible that preterm babies were preferentially protected from the cold, as most mothers provided kangaroo care and breast-fed their babies. It could also be that highly susceptible very preterm babies died of other causes (neonatal sepsis/respiratory distress syndrome/pneumonia were the commonest causes of neonatal death, all potentially related to prematurity).

A limitation of this study is that despite almost 4 years of surveillance, there was a quasi-biennial periodicity to RSV LRTI, which affected the overall rates. However, rates during the RSV season were presented that, for the most part, are double the annual rates. We opted to target babies with severe or very severe LRI as well as hospitalized children for NP swabbing. Hence, we could not calculate the burden of nonsevere RSV LRTIs. Melghat has a high infant mortality, and it could be argued that these data are not generalizable to the rest of India. However, almost 30% of districts in India have as high an infant mortality, and several low-income countries in Africa have as high infant mortality rates. In fact, this study is potentially directly applicable to many such rural populations, where it is very difficult to do such studies.

There are several implications of the study for burden of disease estimates as well as planning for maternal immunization or monoclonal antibody use in rural areas with a high infant mortality such as Melghat. The incidence of RSV LRI appears to increase, as babies get older, and is highest in the second half of the first year of life. Although most very severe RSV LRTIs occur in the first 6 months of life, there is a significant burden of disease later on. RSV mortality is also highest in the first 6 months of life, thus although maternal immunization and birth doses of monoclonal antibodies could potentially prevent mortality, a significant burden of severe disease occurs in older infants and young children and there is a very important place for other immunization strategies to prevent morbidity in these children.

## Supplementary Data

Supplementary materials are available at *Clinical Infectious Diseases* online. Consisting of data provided by the authors to benefit the reader, the posted materials are not copyedited and are the sole responsibility of the authors, so questions or comments should be addressed to the corresponding author.

ciab508_suppl_Supplementary_Material

## Notes

***Acknowledgments.*** The authors thank Niteen Wairagkar, Padmini Srikantiah, and Prachi Vora from the Bill and Melinda Gates Foundation for their input and guidance at every stage of this study.

***Members of the steering committee:*** Prof. Shabir A. Madhi, Dean, Faculty of Health Sciences, University of the Witwatersrand, Johannesburg, South Africa; Dr Sanjay Mehendale, Director Research, PD Hinduja Hospital and Medical Centre, Pune, India, Former Additional Director General, Indian Council of Medical Research New Delhi India; Prof. Sanjay Zodpey, Vice President—Academics, Public Health Foundation of India, New Delhi, Director—Indian Institute of Public Health, Delhi and Dr Subhash R Salunke Senior Advisor, PHFI and Officiating Director, IIPH-Bhubaneswar and former Director General Health Services of Maharashtra State. We thank the MAHAN trust foundation, Dr Kavita Satav, staff and data entry team, all the study team, the supervisors, counsellors, and village health workers who worked tirelessly through the study. Dr Shinde, who was the pediatrician that took care of our study subjects at the MAHAN hospital and was one of the readers of the verbal autopsies. The sincere efforts of the National Influenza Centre technical staff at the National Institute of Virology are acknowledged. We acknowledge with gratitude the assistance of the Hon. District Collector and Additional District Medical Officer Dr Bobade, the pediatricians at the subdistrict hospital, the 93 village Panchayat Heads and Council Members, Dr Shubhada Khirwadakr, Dr Abhijit Bharadwaj, Dr Deoghare, and Dr Patil who taught all our supervisors and counsellors as well as the local traditional healers and auxiliary nurse midwives in grief counselling and provided grief counselling; and all the traditional midwives and auxiliary midwives who assisted with sample collection and grief counselling. We acknowledge Dr Elisabeth Root, Ohio State University, who set up the Geographic Information System at the site and Robert Suss, University of Colorado, for assistance with figures. Finally, and most importantly, we acknowledge with gratitude all of the families and their young children that participated in the study who graciously let us into their homes and allowed us to follow their babies throughout the study and especially those families with babies that died, to whom we could offer no solace, except to know that their participation will help other babies in the future from dying of preventable causes.

***Supplement sponsorship.*** This supplement is sponsored by the Bill and Melinda Gates Foundation.

***Financial support.*** Bill and Melinda Gates Foundation OPP1128468, Caring Friends and Bajaj Holdings and Investment Department.

***Potential conflicts of interest.*** E.S. reports grants, personal fees, and nonfinancial support from Astra Zeneca Inc; grants, personal fees, and nonfinancial support from Merck & Co.; grants, personal fees, and nonfinancial support from Regeneron Inc; grants, personal fees, and nonfinancial support from Pfizer Inc; personal fees, nonfinancial support, and other from Abbvie Inc; personal fees from Alere Inc; grants, personal fees, and nonfinancial support from Roche Inc; other from GSK Inc; grants from Johnson and Johnson; grants and nonfinancial support from Novavax Inc, outside the submitted work. All other authors have no potential conflicts to disclose. All authors have submitted the ICMJE Form for Disclosure of Potential Conflicts of Interest. Conflicts that the editors consider relevant to the content of the manuscript have been disclosed.
