# Supplementary material for: The Burden of Respiratory Syncytial Virus in Children Under 2 Years of Age in a Rural Community in Maharashtra, India
Source: Clin Infect Dis. 2021 Sep 2;73(Suppl 3):S238–47. doi: 10.1093/cid/ciab508 (PMC8411254; doi:10.1093/cid/ciab508)
Supplement: ciab508_suppl_Supplementary_Material [file ciab508_suppl_supplementary_material.docx]

**Supplementary Methods**

1. **Study Design**

Blue dashed line denotes the population used in analyses


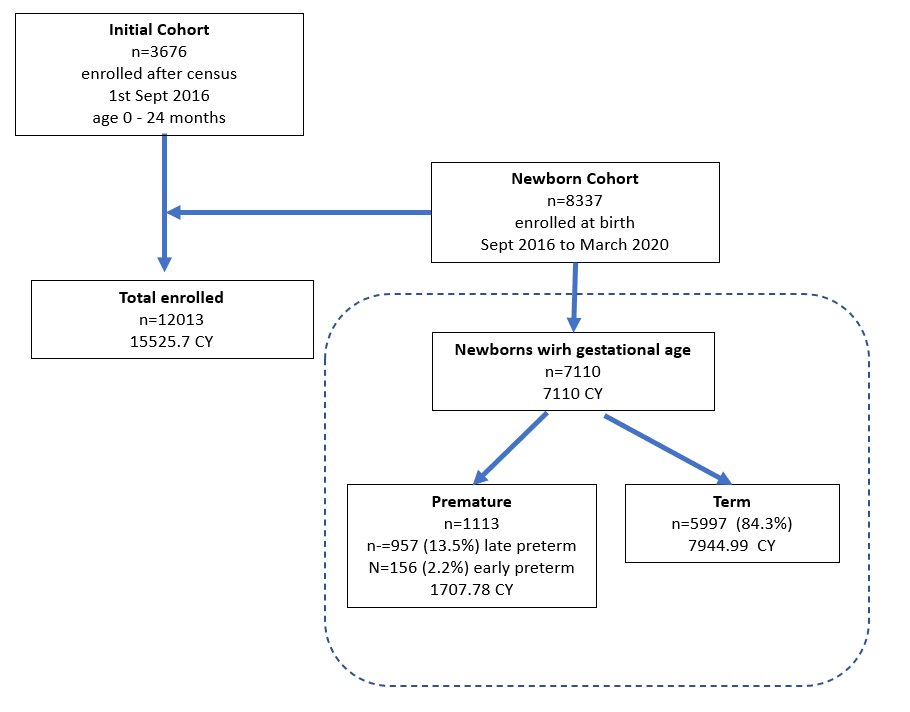


Supplementary Figure 1. Study Design and the Sub Population used in Analyses

1. **Monitoring of Pregnant Women in villages and Estimation of Gestational Age**

The monitoring of pregnancies was performed by Village health workers (VHWs), semiliterate trained local tribal health workers selected by community through community meetings.

The two ways pregnancies in the population were identified in villages were as follows:

1) Regular home visits by the VHWs to women in the age group of 14 to 50 years of age group recording data on their last menstrual period (LMP).

2) Newly married women in villages are identified by door to door visits and LMP information tracked.

By above methods, the VHWs identified early pregnancy in the population. All pregnant women were visited regularly, once a month to monitor blood pressure and weight, and to develop a rapport with them. The VHWs gave advice to pregnant women regarding proper nutrition and on obtaining check-up visits at the hospital or antenatal care clinic. They also gave advise on obtaining medical care to any pregnant women with complications. High risk pregnant women were visited more frequently as needed. The VHWs continued to follow all pregnant women until delivery, when the newborn was enrolled.

**Calculation of Gestation age**

Gestational age by LMP is calculated from the birth date and subtracting the first day of the last menstrual period. As the VHWs recorded data on the last day of the last menstrual period, an adjustment was made of subtracting the last day of menstrual period from the birth date plus seven days. Newborns with calculated gestational age of <168 days (24 weeks) and >308 days (44 weeks) were not included in analysis.

The graph of recorded birth weight and calculated gestational age, shown below, shows the expected relationship for birth data in our newborn cohort.


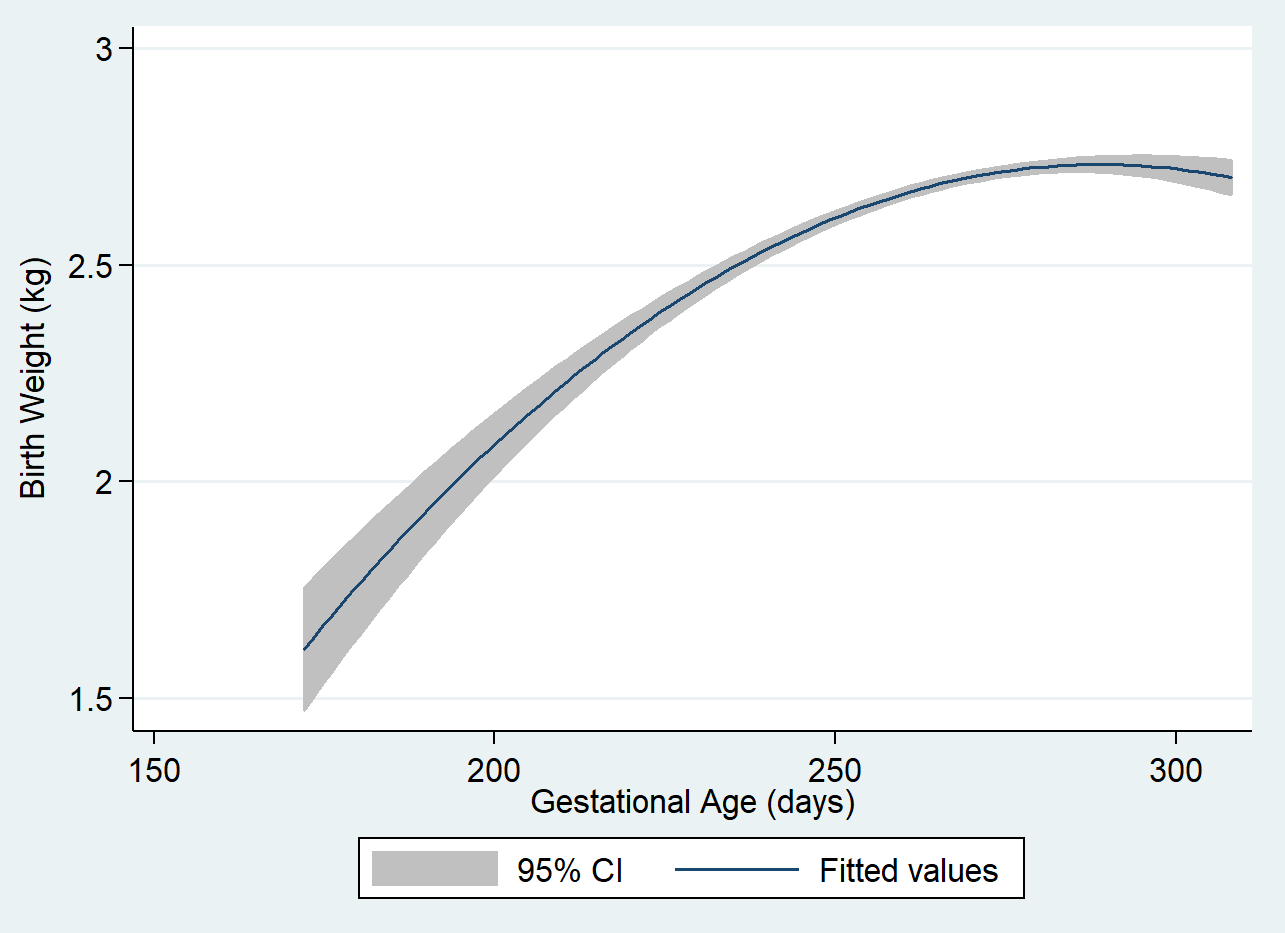


Supplementary Figure 2. Recorded Birth Weight and Gestational Age of Population

1. **Nasopharyngeal swab (NPS) collection and Real Time PCR testing**

Nasopharyngeal swabs were collected from study subjects if chest wall indrawing or general danger signs were present. Samples were collected by the Village Health Workers if the subject met the criteria at the weekly home visits in their assigned village and by Counsellors when a subject presented to a government hospital. Samples were collected using a flocked swab which was placed in a universal viral transport media (UTM) manufactured by Copan Carlsbad, CA 92010, USA. The Copan UTM can be stored at room temperature for 6 months without compromising the quality of sample, hence could be used to collect NPS from remote field areas and transferred to the base hospital at room temperature. This is a replicable way of NPS collection in lower-middle income countries community studies. The NPS were brought to MAHAN hospital and were stored at -8 degree Celsius. Every 15 days, the NP swabs collected were transferred at room temperature to ICMR -National Institute of Virology Pune for testing in batches. The samples were tested for a panel of respiratory viruses including RSV A and RSV B by real time PCR.

Briefly, RNA was extracted using MagMax-96 kit as per manufacturer’s protocol. All the specimens were tested by real-time reverse-transcription polymerase chain reaction (rRT-PCR) for the following viruses: influenza A [A(H1N1)pdm09, A(H3N2)], influenza B [B/Yamagata & B/Victoria] along with house-keeping RNaseP gene (CDC, WHO), respiratory syncytial virus A&B, metapneumovirus, parainfluenza virus 1,2,3,4, rhinovirus and adenovirus using protocol described earlier(1, 2).

Further nucleic acid amplification was performed using one-step reverse transcriptase polymerase chain reaction (qRT-PCR SuperScriptTM III kit, Invitrogen, USA). A 25 µl PCR reaction comprised of 10 µmol of each forward and reverse primer, 5 µmol of TaqMan probe, 12.5 µl 2X buffer, 0.5 µl SuperScriptTM III enzyme and 5 µl nucleic acid templates. Thermal cycling conditions were: 50 ºC for 30 minutes for reverse transcription, initial denaturation at 94 ºC for 5 minutes, 45 cycles of three steps – 15 seconds at 94 ºC, 15 seconds at 50 ºC and 30 seconds at 55°C incubation step during which fluorescence data were collected.

1. **Calculation of Wealth Score using Principal Component Analysis**

An asset-based approach was used to measure household wealth, an approach that was first proposed by Filmer and Pritchett (3). This is increasingly being used to quantity economic status in analyses of health inequalities (4), as it was designed to overcome the challenges in measuring income in low and middle-income countries. In this setting information on income and consumption is often unavailable, unreliable and difficult to measure. It is especially relevant for a population where there is a significant level of self-subsistence agriculture. The ownership of durable goods, can be both representative of longer-term wealth and less sensitive to fluctuations in income.

A wealth index score was generated from the household ownership of durable assets (e.g. car, refrigerator, television), housing characteristics (e.g. dwelling floor and roof material, toilet facilities), and access to services (e.g. electricity supply, drinking water source). A summary measure was created after applying factor weights to each variable. These factor weights were determined through comparison with the rural subset of the Demographic and Health Survey (DHS) national survey data for India 2015-2016 using principal components analysis. In this manner, a wealth index score was found for each household. (3-6)

**Supplementary Results**

**Supplementary Table 1**

| **Supplementary Table 1. Durable Assets and Housing Characteristics of Study Population** | | | | |
| --- | --- | --- | --- | --- |
|  | **Cohort n=7110** | **Term (GA>=37 weeks) n=5997** | **Late preterm (GA <37 >=32 weeks) n=957** | **Early preterm (GA <32 weeks) n=156** |
| **WEALTH SCORE** | -1.3 (SD=1.6) | -1.3 (SD=1.6) | -1.6 (SD=1.5) | -1.7 (SD=1.5) |
| Characteristics | |  |  |  |
| **House ownership** | |  |  |  |
| Own | 7077 (99.5%) | 5969 (99.5%) | 953 (99.6%) | 155 (99.4%) |
| Rent | 19 (0.3%) | 18 (0.3%) | 1 (0.1%) | 0 (0.0%) |
| Other | 13 (0.2%) | 10 (0.2%) | 2 (0.2%) | 1 (0.6%) |
| Unknown | 1 (0.0%) | 0 (0.0%) | 1 (0.1%) | 0 (0.0%) |
|  |  |  |  |  |
| **Electricity** |  |  |  |  |
| yes, metered/own | 2737 (38.5%) | 2332 (38.9%) | 352 (36.8%) | 53 (34.0%) |
| yes, but no meter | 2761 (38.8%) | 2337 (39.0%) | 365 (38.1%) | 59 (37.8%) |
| no electricity | 1611 (22.7%) | 1328 (22.1%) | 239 (25.0%) | 44 (28.2%) |
| Unknown | 1 (0.0%) | 0 (0.0%) | 1 (0.1%) | 0 (0.0%) |
|  |  |  |  |  |
| **Family owns land** | 3631 (51.1%) | 3097 (51.6%) | 450 (47.0%) | 84 (53.8%) |
| Unknown | 1 (0.0%) | 0 (0.0%) | 1 (0.1%) | 0 (0.0%) |
|  |  |  |  |  |
| **Floor material** | |  |  |  |
| Mud/cowdung | 6593 (92.7%) | 5551 (92.6%) | 894 (93.4%) | 148 (94.9%) |
| Wood | 152 (2.1%) | 126 (2.1%) | 26 (2.7%) | 0 (0.0%) |
| Tile | 351 (4.9%) | 310 (5.2%) | 35 (3.7%) | 6 (3.8%) |
| Other | 13 (0.2%) | 10 (0.2%) | 1 (0.1%) | 2 (1.3%) |
| Unknown | 1 (0.0%) | 0 (0.0%) | 1 (0.0%) | 0 (0.0%) |
|  |  |  |  |  |
| **Roof material** | |  |  |  |
| Tin | 4921 (69.2%) | 4193 (69.9%) | 615 (64.3%) | 113 (72.4%) |
| Kavelu | 1432 (20.1%) | 1185 (19.8%) | 225 (23.5%) | 22 (14.1%) |
| Thatch | 510 (7.2%) | 412 (6.9%) | 80 (8.4%) | 18 (11.5%) |
| Wood | 122 (1.7%) | 99 (1.7%) | 21 (2.2%) | 2 (1.3%) |
| Cement | 113 (1.6%) | 97 (1.6%) | 15 (1.6%) | 1 (0.6%) |
| Asbestos | 10 (0.1%) | 10 (0.2%) | 0 (0.0%) | 0 (0.0%) |
| Other | 1 (0.0%) | 1 (0.0%) | 0 (0.0%) | 0 (0.0%) |
| Unknown | 1 (0.0%) | 0 (0.0%) | 1 (0.1%) | 0 (0.0%) |
|  |  |  |  |  |
|  | **Cohort n=7110** | **Term (GA>=37 weeks) n=5997** | **Late preterm (GA <37 >=32 weeks) n=957** | **Early preterm (GA <32 weeks) n=156** |
| **Paint type** |  |  |  |  |
| Chalk | 4436 (62.4%) | 3794 (63.3%) | 545 (56.9%) | 97 (62.2%) |
| None | 2324 (32.7%) | 1895 (31.6%) | 375 (39.2%) | 54 (34.6%) |
| Synthetic | 287 (4.0%) | 263 (4.4%) | 22 (2.3%) | 2 (1.3%) |
| Other | 60 (0.8%) | 43 (0.7%) | 14 (1.5%) | 3 (1.9%) |
| Unknown | 3 (0.0%) | 2 (0.0%) | 1 (0.1%) | 0 (0.0%) |
|  |  |  |  |  |
| **Source of drinking water** | | |  |  |
| Tap water (public) | 3075 (43.2%) | 2605 (43.4%) | 402 (42.0%) | 68 (43.6%) |
| Tap water (own) | 2047 (28.8%) | 1764 (29.4%) | 244 (25.5%) | 39 (25.0%) |
| Hand pump/bore well (public) | 1003 (14.1%) | 829 (13.8%) | 149 (15.6%) | 25 (16.0%) |
| Well (public) | 799 (11.2%) | 647 (10.8%) | 132 (13.8%) | 20 (12.8%) |
| Well (own) | 61 (0.9%) | 53 (0.9%) | 7 (0.7%) | 1 (0.6%) |
| Hand pump/bore well (own) | 55 (0.8%) | 43 (0.7%) | 10 (1.0%) | 2 (1.3%) |
| Water piped | 34 (0.5%) | 29 (0.5%) | 5 (0.5%) | 0 (0.0%) |
| River | 31 (0.4%) | 24 (0.4%) | 6 (0.6%) | 1 (0.6%) |
| lake | 4 (0.1%) | 3 (0.1%) | 1 (0.1%) | 0 (0.0%) |
| Unknown | 1 (0.0%) | 0 (0.0%) | 1 (0.1%) | 0 (0.0%) |
|  |  |  |  |  |
| **Water purification methods used?** | | | |  |
| Chlorination | 4417 (62.1%) | 3687 (61.5%) | 635 (66.4%) | 95 (60.9%) |
| None | 1068 (15.0%) | 894 (14.9%) | 147 (15.4%) | 27 (17.3%) |
| Boiling | 1018 (14.3%) | 897 (15.0%) | 98 (10.2%) | 23 (14.7%) |
| Water filter | 553 (7.8%) | 475 (7.9%) | 69 (7.2%) | 9 (5.8%) |
| Bottled water | 6 (0.1%) | 4 (0.1%) | 2 (0.2%) | 0 (0.0%) |
| Other | 47 (0.7%) | 40 (0.7%) | 5 (0.5%) | 2 (1.3%) |
| Unknown | 1 (0.0%) | 0 (0.0%) | 1 (0.1%) | 0 (0.0%) |
|  |  |  |  |  |
| **Home has a toilet facility** | 1117 (15.7%) | 997 (16.6%) | 108 (11.3%) | 12 (7.7%) |
| Unknown | 1 (0.0%) | 0 (0.0%) | 1 (0.1%) | 0 (0.0%) |
| **Fuel used for cooking** | |  |  |  |
| Wood | 6712 (94.4%) | 5651 (94.2%) | 912 (95.3%) | 149 (95.5%) |
| Cowdung | 41 (0.6%) | 34 (0.6%) | 6 (0.6%) | 1 (0.6%) |
| Kerosene | 3 (0.0%) | 1 (0.0%) | 2 (0.2%) | 0 (0.0%) |
| Gas | 351 (4.9%) | 309 (5.2%) | 36 (3.8%) | 6 (3.8%) |
| Coal | 2 (0.0%) | 2 (0.0%) | 0 (0.0%) | 0 (0.0%) |
| Electricity | 0 (0.0%) | 0 (0.0%) | 0 (0.0%) | 0 (0.0%) |
| Other | 0 (0.0%) | 0 (0.0%) | 0 (0.0%) | 0 (0.0%) |
| Unknown | 1 (0.0%) | 0 (0.0%) | 1 (0.1%) | 0 (0.0%) |
|  | **Cohort n=7110** | **Term (GA>=37 weeks) n=5997** | **Late preterm (GA <37 >=32 weeks) n=957** | **Early preterm (GA <32 weeks) n=156** |
| **Household Disposable Goods** | |  |  |  |
| Color tv | 3197 (45.0%) | 2768 (46.2%) | 371 (38.8%) | 58 (37.2%) |
| Fan | 2905 (40.9%) | 2525 (42.1%) | 326 (34.1%) | 54 (34.6%) |
| Motorbike | 1491 (21.0%) | 1290 (21.5%) | 175 (18.3%) | 26 (16.7%) |
| Watch | 1479 (20.8%) | 1282 (21.4%) | 165 (17.2%) | 32 (20.5%) |
| Bicycle | 823 (11.6%) | 703 (11.7%) | 98 (10.2%) | 22 (14.1%) |
| Cellphone | 775 (10.9%) | 665 (11.1%) | 97 (10.1%) | 13 (8.3%) |
| Water pump | 306 (4.3%) | 275 (4.6%) | 26 (2.7%) | 5 (3.2%) |
| Fridge | 231 (3.2%) | 203 (3.4%) | 26 (2.7%) | 2 (1.3%) |
| Radio | 125 (1.8%) | 111 (1.9%) | 11 (1.1%) | 3 (1.9%) |
| Tractor | 118 (1.7%) | 109 (1.8%) | 8 (0.8%) | 1 (0.6%) |
| Computer | 84 (1.2%) | 71 (1.2%) | 12 (1.3%) | 1 (0.6%) |
| Home phone | 78 (1.1%) | 66 (1.1%) | 9 (0.9%) | 3 (1.9%) |
| Washer | 51 (0.7%) | 43 (0.7%) | 8 (0.8%) | 0 (0.0%) |
| Car | 60 (0.8%) | 48 (0.8%) | 10 (1.0%) | 2 (1.3%) |
|  |  |  |  |  |
| **Livestock** |  |  |  |  |
| Owns bullock cart | 2411 (33.9%) | 2067 (34.5%) | 296 (30.9%) | 48 (30.8%) |
| Owns chickens | 1602 (22.5%) | 298 (5.0%) | 980 (102.4%) | 1129 (723.7%) |
| Owns goats | 1055 (14.8%) | 198 (3.3%) | 713 (74.5%) | 831 (532.7%) |
| Owns cows/buffalo/ox | 1911 (26.9%) | 417 (7.0%) | 1323 (138.2%) | 1535 (984.0%) |

**References**

1. CDC. CDC protocol of realtime RTPCR for influenza A(H1N1) 2009.

2. Koul PA, Mir H, Saha S, Chadha MS, Potdar V, Widdowson MA, et al. Respiratory viruses in returning Hajj & Umrah pilgrims with acute respiratory illness in 2014-2015. Indian J Med Res. 2018;148(3):329-33.

3. Filmer D, Pritchett LH. Estimating wealth effects without expenditure data—or tears: an application to educational enrollments in states of India. Demography. 2001;38(1):115-32.

4. Vyas S, Kumaranayake L. Constructing socio-economic status indices: how to use principal components analysis. Health policy and planning. 2006;21(6):459-68.

5. Fry K, Firestone R, Chakraborty NM. Measuring equity with nationally representative wealth quintiles. Washington DC. 2014.

6. Bassani DG, Corsi DJ, Gaffey MF, Barros AJ. Local distributions of wealth to describe health inequalities in India: a new approach for analyzing nationally representative household survey data, 1992–2008. PloS one. 2014;9(10):e110694.
